# International veterinary epilepsy task force consensus proposal: diagnostic approach to epilepsy in dogs

**DOI:** 10.1186/s12917-015-0462-1

**Published:** 2015-08-28

**Authors:** Luisa De Risio, Sofie Bhatti, Karen Muñana, Jacques Penderis, Veronika Stein, Andrea Tipold, Mette Berendt, Robyn Farqhuar, Andrea Fischer, Sam Long, Paul JJ. Mandigers, Kaspar Matiasek, Rowena MA Packer, Akos Pakozdy, Ned Patterson, Simon Platt, Michael Podell, Heidrun Potschka, Martí Pumarola Batlle, Clare Rusbridge, Holger A. Volk

**Affiliations:** Animal Health Trust, Lanwades Park, Kentford, Newmarket, CB8 7UU Suffolk, UK; Department of Small Animal Medicine and Clinical Biology, Faculty of Veterinary Medicine, Ghent University, Salisburylaan 133, Merelbeke, 9820 Belgium; Department of Clinical Sciences, College of Veterinary Medicine, North Carolina State University, 1052 William Moore Drive, Raleigh, NC 27607 USA; Vet Extra Neurology, Broadleys Veterinary Hospital, Craig Leith Road, Stirling, FK7 7LE Stirlingshire UK; Department of Small Animal Medicine and Surgery, University of Veterinary Medicine Hannover, Bünteweg 9, 30559 Hannover, Germany; Department of Veterinary and Clinical Sciences, Faculty of Health and Medical Sciences, University of Copenhagen, Frederiksberg C, Denmark; Fernside Veterinary Centre, 205 Shenley Road, Borehamwood, SG9 0TH Hertfordshire UK; Centre for Clinical Veterinary Medicine, Ludwig-Maximilians-University, Veterinärstr. 13, 80539 Munich, Germany; University of Melbourne, 250 Princes Highway, Weibee, 3015 VIC Australia; Department of Clinical Sciences of Companion Animals, Utrecht University, Yalelaan 108, 3583 CM Utrecht, The Netherlands; Section of Clinical & Comparative Neuropathology, Centre for Clinical Veterinary Medicine, Ludwig-Maximilians-University, Veterinärstr. 13, 80539 Munich, Germany; Department of Clinical Science and Services, Royal Veterinary College, Hatfield, AL9 7TA Hertfordshire UK; Clinical Unit of Internal Medicine Small Animals, University of Veterinary Medicine, Veterinärplatz 1, 1210 Vienna, Austria; University of Minnesota College of Veterinary Medicine, D426 Veterinary Medical Center, 1352 Boyd Avenue, St. Paul, MN 55108 USA; College of Veterinary Medicine, University of Georgia, 501 DW Brooks Drive, Athens, GA 30602 USA; Chicago Veterinary Neurology and Neurosurgery, 3123 N. Clybourn Avenue, Chicago, IL 60618 USA; Department of Pharmacology, Toxicology and Pharmacy, Ludwig-Maximillians-University, Königinstr. 16, 80539 Munich, Germany; Department of Animal Medicine and Surgery, Veterinary Faculty, Universitat Autònoma de Barcelona, Campus UAB, Bellaterra, 08193 Barcelona, Spain; Fitzpatrick Referrals, Halfway Lane, Eashing, Godalming, GU7 2QQ Surrey UK; School of Veterinary Medicine, Faculty of Health & Medical Sciences, University of Surrey, Guildford, GU2 7TE Surrey UK

**Keywords:** Dog, Seizure, Epilepsy, Idiopathic epilepsy, Diagnosis

## Abstract

This article outlines the consensus proposal on diagnosis of epilepsy in dogs by the International Veterinary Epilepsy Task Force. The aim of this consensus proposal is to improve consistency in the diagnosis of epilepsy in the clinical and research settings. The diagnostic approach to the patient presenting with a history of suspected epileptic seizures incorporates two fundamental steps: to establish if the events the animal is demonstrating truly represent epileptic seizures and if so, to identify their underlying cause. Differentiation of epileptic seizures from other non-epileptic episodic paroxysmal events can be challenging. Criteria that can be used to make this differentiation are presented in detail and discussed. Criteria for the diagnosis of idiopathic epilepsy (IE) are described in a three-tier system. Tier I confidence level for the diagnosis of IE is based on a history of two or more unprovoked epileptic seizures occurring at least 24 h apart, age at epileptic seizure onset of between six months and six years, unremarkable inter-ictal physical and neurological examination, and no significant abnormalities on minimum data base blood tests and urinalysis. Tier II confidence level for the diagnosis of IE is based on the factors listed in tier I and unremarkable fasting and post-prandial bile acids, magnetic resonance imaging (MRI) of the brain (based on an epilepsy-specific brain MRI protocol) and cerebrospinal fluid (CSF) analysis. Tier III confidence level for the diagnosis of IE is based on the factors listed in tier I and II and identification of electroencephalographic abnormalities characteristic for seizure disorders. The authors recommend performing MRI of the brain and routine CSF analysis, after exclusion of reactive seizures, in dogs with age at epileptic seizure onset <6 months or >6 years, inter-ictal neurological abnormalities consistent with intracranial neurolocalisation, status epilepticus or cluster seizure at epileptic seizure onset, or a previous presumptive diagnosis of IE and drug-resistance with a single antiepileptic drug titrated to the highest tolerable dose.

This consensus article represents the basis for a more standardised diagnostic approach to the seizure patient. These recommendations will evolve over time with advances in neuroimaging, electroencephalography, and molecular genetics of canine epilepsy.

**Electronic supplementary material:**

The online version of this article (doi:10.1186/s12917-015-0462-1) contains supplementary material, which is available to authorized users.

## Background

An epileptic seizure is “a transient occurrence of signs due to abnormal excessive or synchronous neuronal activity in the brain” [1] which may manifest in different ways and may be caused by a variety of underlying aetiologies. Epilepsy is defined as a disease of the brain characterized by an enduring predisposition to generate epileptic seizures. This definition is usually practically applied as the occurrence of two or more unprovoked epileptic seizures at least 24 h apart [2].

The term idiopathic epilepsy (IE) has been used in a variety of settings in the veterinary literature and by veterinarians in clinical practice. Analogous with a recently debated proposal for a revised classification by the International League against Epilepsy (ILAE) [3], it has also been proposed that the term idiopathic should be replaced in the veterinary literature [4]. The term genetic epilepsy was therefore introduced to refer to epilepsy occurring as a direct result of a known or strongly suspected genetic defect (or defects) and in which epileptic seizures are the primary clinical sign of the disorder. In general, genetic epilepsies usually have no identifiable structural brain lesions or other neurologic deficits, and have an age-dependent onset. The term unknown epilepsy has been proposed to refer to epilepsy where the underlying cause is unknown [3, 4]. However, a more recent review article discussed how the substitution of the term ‘idiopathic’ with ‘genetic’ may be misleading and idiopathic epilepsy was defined as an epilepsy of predominantly genetic or presumed genetic origin in which there were no gross neuroanatomic or neuropathologic abnormalities nor other relevant underlying diseases [5]. In our consensus proposal on classification and terminology (see consensus on epilepsy definition, classification and terminology in companion animals) we have explained why we recommend retaining the term IE, and have defined IE as a disease in its own right, *per se.* A genetic origin of IE is supported by genetic testing (when available) and a genetic influence is supported by a high breed prevalence (>2 %), genealogical analysis and/or familial accumulation of epileptic individuals. However in the clinical setting IE remains most commonly a diagnosis of exclusion following diagnostic investigations for causes of reactive seizures and structural epilepsy.

To date different criteria have been used in the veterinary literature to diagnose IE. The majority of veterinary studies have used a history of recurrent epileptic seizures, an unremarkable inter-ictal clinical and neurological examination and an unremarkable complete blood cell count and serum biochemistry profile as the minimum criteria for its diagnosis. However, the exact parameters included in the biochemistry profile vary among studies and institutions. Age at seizure onset has not been consistently used as a diagnostic criteria, and when used the age range has varied, most commonly being 1 to 5 years, 6 months to 5 years or 6 months to 6 years. An unremarkable magnetic resonance imaging (MRI) study of the brain and cerebrospinal fluid (CSF) analysis have been used inconsistently as diagnostic criteria and there has been wide variability in MRI protocols. To further support the diagnosis of IE, particularly when brain MRI was not performed, a minimum follow-up period ranging from 1 to 3 years without the development of inter-ictal neurological deficits has also been suggested [6–8].

To improve consistency in the diagnosis of IE amongst institutions and clinical studies we have produced the following consensus proposal.

### Criteria for the diagnosis of epileptic seizures

The diagnostic approach to the patient presenting with a history of suspected epileptic seizures incorporates two fundamental steps:

1. Establish if the events the animal is demonstrating truly represent epileptic seizures or are consistent with a different episodic paroxysmal disorder.
2. Identify the underlying cause of the epileptic seizure.

#### 1. Is the animal having epileptic seizures?

First of all the clinician needs to determine whether the dog is indeed having epileptic seizures. A detailed and accurate history is the foundation for investigation of the seizure patient [9]. The owner of the epileptic dog should complete a standardised epilepsy questionnaire (Additional file 1) and obtain video-footage whenever possible. This information can help the clinician to clarify the nature of the event (*e.g.,* epileptic seizure versus other episodic paroxysmal event) and its phenotype. Numerous disorders can result in episodic paroxysmal events that may mimic epileptic seizures. A detailed review of paroxysmal movement disorders as well as other events which may mimic epileptic seizures is beyond the scope of this consensus article and can be found elsewhere [10, 11]. The main focus of this section of our consensus article is the criteria allowing differentiation of epileptic seizures from other non-epileptic episodic paroxysmal events (Table 1).

**Table 1:** Clinical characteristics of episodic disorders

Discriminator	Syncope	Narcolepsy/Cataplexy	Neuromuscular weakness	Paroxysmal behaviour changes (compulsive disorder)	Vestibular attack	Paroxysmal Dyskinesia	Idiopathic head tremor	Seizure
Clinical status between episodes	Normal or arrhythmia, pulse deficits, heart murmur, cyanosis, abnormal lung auscultation	Altered sleep/wake cycle, normal clinical examination	Normal or generalised weakness, muscle atrophy, pain, decreased reflexes	Normal	Normal	Normal	Normal	Normal or forebrain signs
Precipitating event or trigger	Exercise, excitement	Excitement, eating	Activity, exercise	Behavioural triggers (*e.g.,* fear)	None	None or activity, exercise, excitement, stress	None or stress, fatigue, overstimulation	None or flashing lights, anxiety, stress
Pre-event changes	None	None	None	None	None	None	None	Pre-ictal signs may be observed including: anxiety, restlessness, increased affection, contact-seeking, withdrawal, hiding, aggressiveness, and vocalization
Event description	Brief, sudden collapse and rapid recovery	Sudden collapse	Stiff, stilted gait prior to collapse	Pacing, barking, licking, chasing imaginary objects or tail, chewing objects	Head tilt, nystagmus, vestibular ataxia, collapse towards side of head tilt	Dystonia, chorea, ballismus, athetosis, tremors, impaired posture, inability to stand or walk	Vertical or horizontal rhythmic head movement	Depending on seizure focus, focal or generalized, tonic-clonic movements most common
Level of consciousness	Reduced to absent	Normal if only cataplexy. Absent (asleep) in narcolepsy	Normal	Normal	Normal or disorientated	Normal	Normal	Often impaired
Autonomic signs	Possible abnormalities of heart rate and rhythm	None	None	None	None	None	None	Possible: hypersalivation, defaecation, urination
Muscle tone	Flaccid (all body)	Flaccid (all body)	Often flaccid (can appear spastic with certain myopathies)	Normal	Unilateral decrease in extensor muscle tone	Hypertonicity (focal or generalised)	Normal	Typically increased: tonic (hypertonicity) or alternating tonic-clonic movements
Lateralising signs	No	No	No	No	Yes	Possible	No	Possible
Duration	Seconds	Seconds to minutes	Minutes to hours	Minutes to hours	Seconds to hours	Seconds to hours	Seconds to hours	Seconds to minutes or > 5 min in case of status epilepticus
Post-episodic changes	None	None	None	None	None	None or tiredness	None, tiredness, or restlessness	Post-ictal signs frequently occur including: disorientation, aggressive behaviour, restlessness, pacing, lethargy, deep sleep, hunger, thirst, ataxia, proprioceptive deficits, and blindness
Further comments	May be accompanied by cough, increased respiratory noise	Often occurs in young purebred dogs.	May be accompanied by dysphagia, dysphonia, regurgitation, dyspnoea	History of anxiety disorder	Subtle signs of vestibular disease might persist	Interaction with the owner can alleviate or interrupt the episode. Consider breed specific disorders and age at onset.	Episodes can be interrupted by the owner	Facial muscles often involved during the ictus

A complete clinical and neurological examination may help identify abnormalities suggestive of underlying disease processes, including cardiovascular system abnormalities in dogs with syncope and clinical signs of neuromuscular disease, vestibular dysfunction or forebrain disease.

Paroxysmal movement disorders or paroxysmal dyskinesias refer to abnormal, sudden, involuntary contraction of a group of skeletal muscles which recur episodically [10]. These paroxysms can be challenging to differentiate from epileptic seizures, particularly from focal motor epileptic seizures. Animals affected by movement disorders are often normal between episodes. The absence of other clinical signs during the episodes, including autonomic signs, changes in consciousness and electroencephalographic abnormalities, have been suggested to support the diagnosis of paroxysmal movement disorders [10]. However, focal epileptic seizures can occur with no concurrent alteration in consciousness or autonomic signs and electroencephalography (EEG) is often challenging to perform in the clinical setting. In a recent study evaluating the diagnostic utility of inter-ictal short time EEG recordings in epileptic dogs under general anaesthesia with propofol and the muscle relaxant rocuronium bromide, interictal paroxysmal epileptiform activity was detected in only 25 % of IE dogs [12]. The signalment and age at onset of the paroxysmal event can assist in establishing the nature of these events. Certain movement disorders are breed-specific, generally occur in young dogs and their phenotype may be well characterised [10]. To date the associated genetic defect (*e.g.,* deletion in the gene *BCAN*) has been identified only in Cavalier King Charles spaniels with paroxysmal exercise-induced dyskinesia (also known as episodic falling) [13, 14]. Genetic investigations in other breeds are ongoing. Identification of causative genetic mutations of breed specific movement disorders will significantly improve our ability to diagnose these conditions. Interestingly, specific mutations in human patients with dyskinesias may also be associated with epileptic seizures or a high occurrence of seizure disorders in their relatives [15].

A genetic predisposition to IE has been suggested in numerous canine breeds [16] and a familial history of recurrent epileptic seizures or IE should raise the suspicion of IE, although diagnostic procedures need to be performed to exclude other aetiologies. Generalised epileptic seizures typically occur at rest or during sleep, last less than 5 min and are usually followed by abnormal clinical manifestations (post-ictal signs) including disorientation, restlessness, pacing, lethargy, deep sleep, hunger, thirst, ataxia, proprioceptive deficits, and less commonly, aggressive behaviour and blindness. The presence of impaired consciousness (*e.g.,* altered awareness and responsiveness to the environment and stimuli), oro-facial muscle involvement, autonomic signs and convulsions during the ictus all support the classification of the episodes as epileptic seizures. During the ictus (particularly during the generalized epileptic seizure phase) the animal cannot be distracted and the owner cannot alter the course of the event by manipulating the dog. Conversely, dogs with paroxysmal movement disorders tend to continue to attempt to perform the activity they were previously doing (*e.g.,* playing) during the paroxysmal event and owner intervention may alter the course of the episode. For example, in the majority of Dobermanns with idiopathic head tremor, the owners reported that they could consistently interrupt each head tremor episode. In some cases, stroking the dogs, talking to them, or asking them to get up was sufficient to interrupt the episode. In other cases, stronger stimuli (favourite toys or snacks, encouraging them, taking them for a walk) were needed to interrupt the head tremor episode [17]. Similarly in a study in English bulldogs with idiopathic head tremors, several owners reported that distraction or treats were generally sufficient to alter or stop the episodes [18].

A recent study highlighted the challenge in differentiating epileptic and non-epileptic paroxysmal events. This study investigated the level of agreement between veterinarians (both neurology specialists and non-specialists) in the description and classification of videos depicting canine and feline paroxysmal events, where the observers were blinded to the history, results of diagnostic investigations and treatment response [19]. The level of agreement on whether a paroxysmal event was an epileptic seizure or other paroxysm was fair. Overall agreement on epileptic seizure type was moderate. Generalised epileptic seizures had the highest level of agreement and focal epileptic seizures had the lowest. Agreement was fair for level of consciousness and the presence of autonomic signs, but poor for neurobehavioral signs. Agreement for motor signs ranged from poor to moderate. There were significant differences in epileptic seizure semiology and classification between specialists and non-specialists.

Absolute confirmation of the epileptic nature of an event can only be obtained by observing simultaneously the characteristic EEG changes and physical manifestation of the seizures, however this is rarely practical in veterinary medicine and currently there is no reliable, standard protocol for acquiring EEG recordings in dogs. Physiological artifacts (*e.g.,* muscle contractions, electrocardiogram, electrooculogram) and physical factors (*e.g.,* EEG instrumentation, electrode type and montage, methods of patient restraint) affect acquisition and interpretation of EEG tracings [20]. Variability in the physical factors mentioned above has contributed to discrepancies in the results of numerous veterinary studies evaluating EEG. Efforts are currently in progress to further develop EEG recording in veterinary clinical practice. Although it is unlikely that EEG will become a routine diagnostic procedure for all epileptic dogs in the near future, EEG may become more widely used by veterinary neurology specialists for the investigation of selected cases (*e.g.,* dogs in which a diagnosis of epilepsy versus other episodic paroxysmal disorder is particularly challenging). As an example, a veterinary video-EEG study diagnosed a juvenile Chihuahua with subtle myoclonic absence events with perioral myoclonia and head twitching [21]. The author identified bilateral generalised synchronous 4Hz spike-and-wave complexes on ictal EEG time locked with the "absence-like" event, along with rhythmically correlated head and nose twitching. In this case video-EEG was essential to confirm the epileptic nature of the episodes. Currently the paucity of veterinary literature does not allow a clear consensus recommendation for EEG recording in veterinary patients to be proposed.

#### 2. What is the cause of the epileptic seizure?

After having established that the episodic paroxysmal events do indeed represent epileptic seizures, the next step is to determine the underlying cause as this will have major implications on treatment selection and prognosis. Both intra and extra cranial disorders can cause seizure activity.

##### Reactive seizures

Reactive seizures can result from systemic metabolic disorders (*e.g.,* hypoglycaemia, electrolyte disorders, portosystemic shunt resulting in hepatic encephalopathy) or from intoxications (*e.g.,* carbamates, organophosphates, lead poisoning, ethylene glycol toxicity, metaldehyde, strychnine). The history and clinical presentation may help the clinician to suspect a particular aetiology, although diagnosing certain intoxications can be quite challenging. In a recent study the most frequent cause of reactive seizures were intoxications (39 %, 37/96 of dogs) and hypoglycaemia (32 %, 31/96 of dogs) [22]. In this study, 41 % (39/96) of dogs were presented in status epilepticus [22]. Another study showed that dogs with reactive seizures caused by exogenous toxicity have a significantly higher risk of developing status epilepticus, particularly as first manifestation of a seizure disorder, than dogs with other seizure aetiologies [23]. Dogs with poisoning had a 2.7 times higher risk of presenting in status epilepticus at seizure onset than dogs with IE or structural epilepsy [23]. The clinical presentation in dogs with metabolic and toxic disorders is variable and depends on the underlying aetiology. Toxic disorders often have an acute (< 24 h) onset and neurological signs may be preceded or accompanied by gastrointestinal, cardiovascular or respiratory signs. Dependent on the specific toxin, muscle tremors and fasciculations are frequently the initial clinical signs. Metabolic disorders can present with an acute, subacute, or chronic onset and may be progressive or relapsing and remitting. For example, chronic lead intoxication may result in recurrent seizures. Systemic clinical abnormalities can often be detected on general physical examination. Generally neurological examination reveals deficits consistent with diffuse, bilateral and often symmetrical forebrain involvement.

##### Structural epilepsy

Structural forebrain disorders resulting in epileptic seizures include a large array of conditions including vascular, inflammatory/ infectious, traumatic, anomalous/ developmental, neoplastic and degenerative diseases. Neurological examination is often abnormal and may reveal asymmetric neurological deficits in dogs with lateralised brain pathology. In a recent study, 47 % of dogs with lateralised structural cerebral lesions had asymmetrical neurological deficits and 55 % of dogs with symmetrical structural brain lesions had symmetrical neurological deficits identified on neurological examination [24]. Dogs with inter-ictal neurological abnormalities were 16.5 times more likely to have an asymmetrical structural cerebral lesion and 12.5 times more likely to have a symmetrical structural cerebral lesion than IE [24]. A normal inter-ictal neurological examination, however, does not completely rule out structural epilepsy as focal lesions in particular areas of the forebrain, such as the olfactory bulb, frontal and pyriform lobes (“clinically silent regions”) can result in epileptic seizures without any other neurological signs. Indeed, in the study mentioned above, 23 % (34/146) of dogs with structural epilepsy had a normal neurological examination in the inter-ictal period. In a study on risk factors for development of epileptic seizures in dogs with intracranial neoplasia, an epileptic seizure was the first sign of intracranial disease noted by the owners in 76 % of dogs and dogs with frontal lobe neoplasia were more likely to develop epileptic seizures than dogs with neoplasia in other intracranial locations [25].

The inter-ictal neurological status has been combined with the dog’s age at epileptic seizure onset in an attempt to predict the probability of identifying structural cerebral disorders in dogs presenting with recurrent epileptic seizures (see section below on recommendation on when to perform MRI of the brain).

Epileptic seizure type (*e.g.,* focal versus generalised) should not be used as an isolated variable to predict the presence of structural cerebral disease. Indeed focal epileptic seizures have been reported in dogs with IE [26–29] and in a recent study the prevalence of generalised epileptic seizures was similar between dogs with IE (77 %) and dogs with asymmetrical structural cerebral lesion (79 %) [24]. Furthermore, in a study in dogs with epileptic seizures associated with intracranial neoplasia, 93 % of dogs had generalised epileptic seizures and 7 % had focal epileptic seizures [25]. A detailed description of diagnosis of exogenous toxic, metabolic and structural forebrain disorders is beyond the scope of this consensus article and can be found elsewhere [30–32].

##### Idiopathic epilepsy

The diagnosis of IE is one of exclusion and is made based on the age at epileptic seizure onset, unremarkable inter-ictal physical and neurological examinations, and exclusion of metabolic, toxic and structural cerebral disorders by means of diagnostic investigations. A history of IE in genetically related dogs further supports the diagnosis.

The dog’s age range at seizure onset has been evaluated in various studies in order to predict the likelihood of diagnosing IE (see *recommendation on when to perform MRI of the brain*).

### Criteria for the diagnosis of Idiopathic epilepsy

#### Tier I confidence level for the diagnosis of IE

A history of two or more unprovoked epileptic seizures occurring at least 24 h apart, age at epileptic seizure onset of between 6 months and 6 years, unremarkable inter-ictal physical and neurological examination (except for antiepileptic drug (AED) induced neurologic abnormalities and post-ictal neurologic deficits), and no clinically significant abnormalities on minimum data base (MDB) blood tests and urinalysis. MDB blood tests include: complete blood cell count (CBC), serum biochemistry profile (sodium, potassium, chloride, calcium, phosphate, alanine aminotransferase (ALT), alkaline phosphatise (ALP), total bilirubin, urea, creatinine, total protein, albumin, glucose, cholesterol, triglycerides, and fasting bile acids and/or ammonia). Urinalysis includes specific gravity, protein, glucose, pH, and sediment cytology. A family history of IE further supports the diagnosis.

Dogs with suspected AED-induced neurologic abnormalities and/ or postictal neurologic deficits should be re-examined when steady state serum concentrations of AED is achieved or resolution of post-ictal changes is expected (within less than 1 week), respectively.

Neurobehavioural comorbidities can occur in dogs with IE [33], similarly to human patients [34], and their presence should therefore not imply a diagnosis of structural epilepsy. However MRI studies of the brain (see consensus statement on epilepsy-specific brain MRI protocol) and CSF analysis are recommended in these dogs.

Additional discretionary laboratory parameters depending on the index of disease suspicion include: fasting and post-prandial bile acids, fasted ammonia and abdominal ultrasound when hepatic encephalopathy is suspected; total T4 (TT4), free T4 (fT4), and thyroid stimulating hormone (TSH) when thyroid disorders are suspected (thyroid testing should be performed prior to long term treatment with AEDs due to possible interactions between AED and the thyroid hormones); fructosamine, glucose curve and/ or glucose:insulin ratio when insulinoma is suspected; serum creatine kinase (CK) activity and lactate levels whenever muscle disease is suspected (results should be interpreted in relation to time of sampling since the last epileptic seizure event and severity and duration of the epileptic seizure event, as excessive muscle activity during epileptic seizures activity can transiently increase CK activity and lactate levels); serology/ polymerase chain reaction (PCR)/ antigen testing for regional infectious disorders (these should be performed whenever infectious disorders are suspected); vitamin B12 when cobalamin malabsorption is considered; ionized calcium when hypocalcemia is suspected; testing for specific toxins or toxicological screening by mass spectroscopy when toxin exposure is suspected; quantification of amino acids and organic acids and determination of glycosaminoglycans, oligosaccharides, purines, and pyrimidines in serum, CSF or urine when inborn errors of metabolism are suspected; genetic testing when a disorder with known genetic mutation is suspected (*e.g.,* benign familial juvenile epilepsy in the Lagotto Romagnolo, progressive myoclonic epilepsy in miniature wire haired Dachshunds, L-2-hydroxyglutaric aciduria in Staffordshire bull terriers). In addition, imaging of the thorax and abdomen should be performed when metastatic neoplastic disease is a possibility. Ocular fundic examination and non-invasive blood pressure measurement should also be performed when hypertension is suspected. Further details on diagnostic investigations to identify underlying aetiologies of seizures can be found elsewhere [30].

#### Tier II confidence level for the diagnosis of IE

Unremarkable fasting and post-prandial bile acids, MRI of the brain (see consensus statement on epilepsy-specific brain MRI protocol) and CSF analysis in addition to factors listed in tier I.

If abnormalities compatible with seizure-associated changes are identified on MRI, the MRI protocol should be repeated after a 16 week seizure free interval (whenever possible) (see below: *Epileptic seizure-associated CSF and brain MRI changes*).

If the results of routine CSF analysis are abnormal then additional testing on CSF and serum for regional infectious disorders should be performed. CSF abnormalities (generally mild) may occur as a result of epileptic seizure activity [35] (see below: *Epileptic seizure-associated CSF and brain MRI changes*). Time to resolution of epileptic seizure-associated CSF abnormalities is unknown. If CSF abnormalities are present but the results of investigations for infectious disorders on CSF and serum are negative and brain MRI is unremarkable or shows post-ictal changes, then the CSF analysis should be repeated following a seizure free interval of at least 6 weeks.

#### Tier III confidence level for the diagnosis of IE

Identification of ictal or inter-ictal EEG abnormalities characteristic for seizure disorders according to criteria validated in human medicine, in addition to factors listed in tier I and II. However, further research is needed to characterise the optimal protocol for EEG use in clinical veterinary practice.

##### Epileptic seizure-associated CSF and brain MRI changes

Epileptic seizure activity has been reported to cause CSF abnormalities [35] and intraparenchymal cerebral signal changes on MRI performed within 14 days of the last epileptic seizure [36]. The MRI signal changes are located unilaterally or bilaterally, predominantly in the piriform and temporal lobes, and sometimes also in the olfactory bulb and frontal lobe. The signal changes are characterised by varying degrees of hyperintensity on T2 weighted, FLAIR and diffusion-weighted imaging, hypointensity on T1 weighted images, and occasionally heterogenous contrast enhancement following gadolinium administration [36, 37]. Following antiepileptic treatment only, these signal changes partly or completely resolved on repeated MRI 10 to 16 weeks later, indicating that these changes most likely represent cytotoxic and vasogenic oedema induced by the epileptic seizures. Histologic examination of the affected temporal cortex, hippocampus and piriform lobe revealed oedema, neovascularization, reactive astrocytosis, and acute neuronal necrosis [36]. Repeated MRI of the brain after a period of seizure control, along with clinical and CSF analysis findings, may help to differentiate epileptic seizure-induced changes from inflammatory or neoplastic epileptogenic structural lesions [36].

Mild postictal CSF pleocytosis and sometimes also increased protein concentration have been reported as a transient CSF abnormality in people, generally following repetitive generalized tonic-clonic seizures [38]. Mild CSF pleocytosis (up to 12 WBC/μl, reference range 0–5 WBC/μl) has also been identified following single focal or generalized tonic-clonic seizures in a small number of patients, particularly when CSF sampling occurred within 12 h of the last seizure [39]. A study in idiopathic epileptic dogs identified an association between CSF white blood cell (WBC) count and time interval between the last seizure and the collection of the CSF. The longer the time interval, the lower the CSF WBC count. However, the CSF WBC count was within the reference range (≤5 WBC/μl) in all dogs and 80 % of dogs underwent CSF sampling 3 or more days after the last seizure. No association was found between CSF protein concentration and time of CSF collection and the occurrence of cluster seizures was not associated with any significant change in CSF WBC or protein concentration [35]. The pathophysiology of seizure-induced CSF pleocytosis remains unclear. It is possible that a transient disturbance of the blood–brain barrier function (which has been demonstrated after seizures in experimental animals) and release of chemotactic substances into the CSF during the seizures result in these CSF abnormalities [40]. Repeated CSF sampling after a seizure free interval reveals no abnormalities [38].

##### Recommendation on when to perform MRI of the brain

The dog’s age at seizure onset and the presence of interictal neurological abnormalities have been evaluated in an attempt to predict the probability of identifying structural cerebral disorders in epileptic dogs. In a study in a non-referral canine population, structural epilepsy was statistically more probable in dogs <1 year or > 7 years of age at seizure onset, whereas IE was statistically more probable in dogs aged 1 to 5 years at first seizure and when the interictal period was longer than 4 weeks [41]. In a retrospective study on a referral population of 240 dog with epileptic seizures, seizure onset between 1 and 5 years of age was associated with a 3.25 times greater likelihood for idiopathic epilepsy than structural epilepsy and reactive seizures [6]. One study reported brain MRI abnormalities in 22 % (14/63) and 90 % (47/52) of epileptic dogs with normal and abnormal neurological examination, respectively [42]. Results of CSF analysis (normal versus abnormal) were significantly associated with the results of the MRI study (normal versus abnormal), in dogs with both normal and abnormal neurological examination [42]. Another study reported clinically significant MRI abnormalities, including olfactory or frontal lobe neoplasia, in 2.2 % (1/46) and 26.7 % (8/30) of inter-ictally normal epileptic dogs younger and older than 6 years of age, respectively [43]. In a study including dogs whose first seizure occurred below the age of one year, 26 % (6/23) of dogs with a normal neurological examination had an underlying structural brain disease identified with MRI and CSF analysis [44]. Another study including dogs whose first seizure occurred ≥7 years of age identified an underlying CNS structural disease in 59 % (53/90) of dogs with an unremarkable inter-ictal neurologic examination [45]. A retrospective study including 99 dogs ≥ 5 years of age at epileptic seizure onset reported that an abnormal neurologic examination had 74 % sensitivity and 62 % specificity to predict structural epilepsy with positive and negative predictive values of 79 %, and 55 %, respectively [46]. Of the 53 dogs with an abnormal neurological examination, 42 (79 %) had a lesion detected by MRI or had abnormal findings on CSF analysis (some dogs had both CSF and MRI abnormalities). Fifteen of the 33 (45 %) dogs with normal neurological examination had structural epilepsy diagnosed on the basis of MRI or CSF analysis results [46]. Another recent study demonstrated that age at seizure onset and neurological examination findings were both significantly associated with type of brain disease (functional versus structural) [24]. In this study, 89 % (230/258) of dogs with IE had an age at seizure onset < 6 years and 84 % (217/258) of dogs with IE were neurologically normal inter-ictally. Dogs that were older at seizure onset were significantly more likely to have an asymmetrical structural cerebral lesion (mean age at seizure onset 7.6 ± 3.4 years) than IE (3.3 ± 2.1 years). The odds of identifying an asymmetrical structural cerebral lesion rather than IE increased 1.6-fold with each additional year of age at seizure onset. Dogs with neurological abnormalities inter-ictally were 16.5 times more likely to have an asymmetrical structural cerebral lesion and 12.5 times more likely to have a symmetrical structural cerebral lesion than IE. Dogs with single seizures rather than cluster seizures were more likely to have IE than an asymmetrical structural cerebral lesion [24]. In another study, of 51 dogs presenting with status epilepticus as the first manifestation of seizure disorder, 45.1 % had structural epilepsy, 31.4 % had reactive seizures and 23.5 % had IE [23]. Dogs with IE had a reduced risk of developing status epilepticus at seizure onset compared to dogs with structural epilepsy or reactive seizures [23].

**Figure 1:**
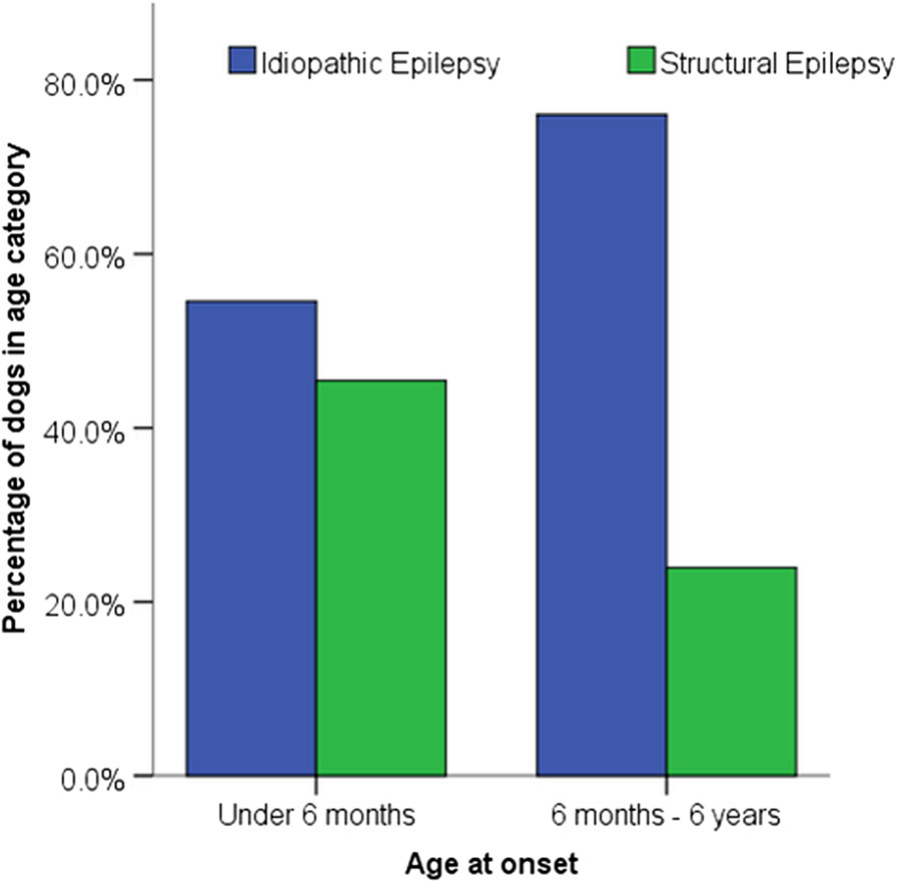
Proportion of dogs with idiopathic and structural epilepsy stratified by age at epileptic seizure onset (< 6 months versus 6 months to 6 years)

**Figure 2:**
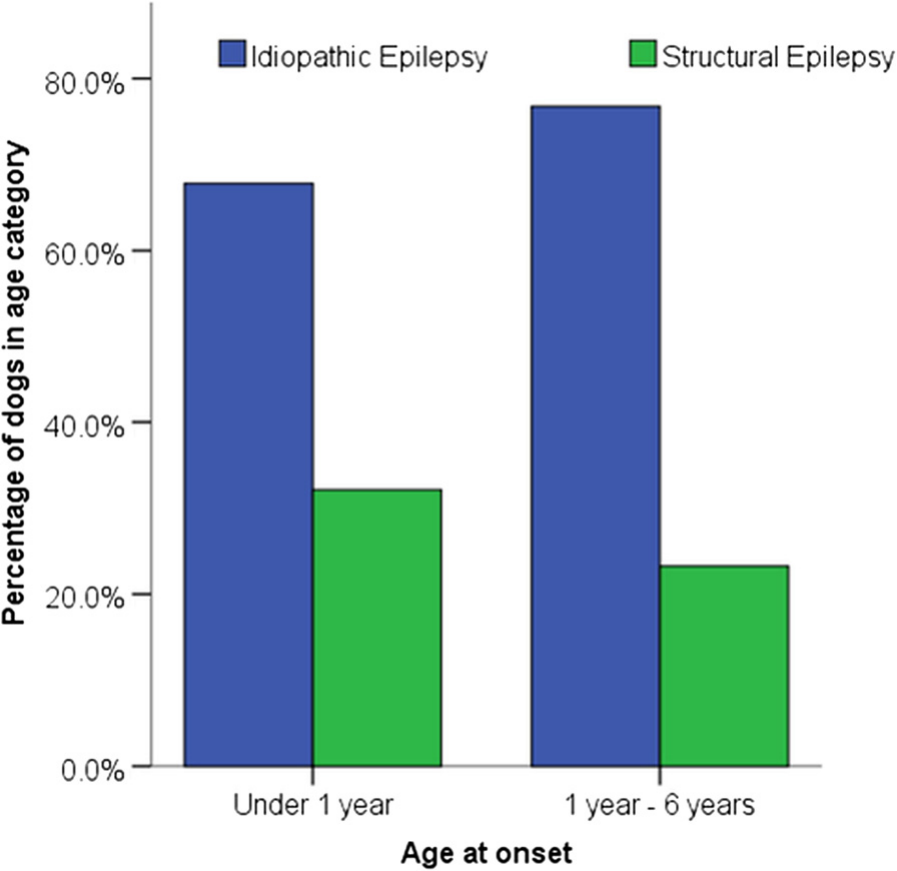
Proportion of dogs with idiopathic and structural epilepsy stratified by age at epileptic seizure onset (< 1 year versus 1 to 6 years)

To further investigate the predictive value of age at epileptic seizure onset to differentiate between idiopathic and structural epilepsy, the data from the studies performed by Pakozdy [6] and Armaşu [24] have been combined and analysed. There were 372 dogs with IE and 236 dogs with structural epilepsy. There was a significant association between age of onset and cause of epilepsy for dogs under 6 years of age at epileptic seizure onset (Chi-squared = 5.136, n = 431, p = 0.023) when the cut-off was set at 6 months (Fig. 1). Dogs between 6 months and 6 years were significantly more likely to be affected by idiopathic than symptomatic epilepsy compared to dogs under 6 months. Whereas, there was no significant association between age of onset and cause of epilepsy for dogs under 6 years of age at epileptic seizure onset (Chi-squared = 2.95, n = 431, p = 0.086) when the cut-off was set at 1 year (Fig. 2). A binary logistic regression demonstrated that dogs aged between 6 months and 6 years at epileptic seizure onset were 2.65 times more likely to be affected by IE than SE (p = 0.03) than those under 6 months of age at epileptic seizure onset. Whereas, a binary logistic regression demonstrated that there was no significant association between age of onset and cause of epilepsy for dogs under 6 years of age at epileptic seizure onset (p > 0.05) when the cut-off was set at 1 year. When comparing the 5 versus 6 years of age at epileptic seizure onset as upper cut off, the 6 year cut off was a better predictor (77.3 % accuracy versus 74.5 %) and had a better model fit with a lower Akaike Information Criteria (AIC) value. A binary logistic regression demonstrated that dogs under 6 at age at epileptic seizure onset were 10.89 times more likely to be affected by IE than structural epilepsy (p < 0.001). Whereas, a binary logistic regression demonstrated that dogs under 5 of age at epileptic seizure onset were 8.00 times more likely to be affected by IE than structural epilepsy (p < 0.001).

Based on the information described above, the authors’ recommendation is to perform MRI of the brain (using the veterinary epilepsy-specific MRI protocol) and routine CSF analysis, after exclusion of reactive seizures, in dogs with:

- age at epileptic seizure onset <6 months or >6 years
- interictal neurological abnormalities consistent with intracranial neurolocalisation
- status epilepticus or cluster seizure
- a previous presumptive diagnosis of IE and drug-resistance with a single AED titrated to the highest tolerable dose.

## Conclusions

The recommendations presented in this article represent the basis of a more standardised diagnostic approach to the seizure patient. These guidelines are likely to evolve over time with advances in structural and functional neuroimaging, EEG, and molecular genetics of canine epilepsy.

## Additional file

Additional file 1:
**Standardised epilepsy investigation questionnaire.**

## Abbreviations

IE: Idiopathic epilepsy
ILAE: International League Against Epilepsy
MRI: Magnetic resonance imaging
CSF: Cerebrospinal fluid
EEG: Electroencephalography
AED: Antiepileptic drug
MDB: Minimum data base
CK: Creatine kinase
PCR: Polymerase chain reaction
