# Supplementary material for: International veterinary epilepsy task force consensus proposal: diagnostic approach to epilepsy in dogs
Source: BMC Vet Res. 2015 Aug 28;11:148. doi: 10.1186/s12917-015-0462-1 (PMC4552251; doi:10.1186/s12917-015-0462-1)
Supplement: Additional file 1: — Standardised epilepsy investigation questionnaire. [file 12917_2015_462_MOESM1_ESM.doc]

**International Veterinary Epilepsy Task Force Epilepsy Questionnaire**

| **Case No.:** | |  | | **Name:** | |  |  |
| --- | --- | --- | --- | --- | --- | --- | --- |
| **Breed:** |  | | | **Name of animal:** |  | |  |
| **Sex:** | M / F | | | **Reproductive status:** | **Entire / Neutered** | |  |
| **Age: Diet:** |  | | | **Body Weight:** |  | |  |
| **Date:………………** | | |  | | | | |
| **There are many possible causes of the episodes that your pet is experiencing, and unfortunately disorders of several body systems can all cause very similar signs. Please take the time to fill in this questionnaire, as the information it contains can be very helpful for us in interpreting the events you are witnessing, and thereby help us to focus our diagnostic investigations.**  **If several options are available, please select all that are appropriate.**  **The word “fit” in this questionnaire refers to episodes your pet is experiencing.** | | | | | | | |

**Regarding your pet’s history:**

**Did your pet have birth complications?** yes no not sure

**Has your pet ever sustained head trauma?**  yes no not sure

**Has your pet ever had meningitis or an infection that** yes no not sure

**involved the brain, spine or nerves?**

**Does your pet suffer from any of the following?** liver disease low blood sugar

kidney disease low potassium

low calcium

**Do you know of any relatives of your pet that suffer**  yes no not sure

**from epilepsy?**

**If yes, please provide a copy of the pedigree to us**

**How old was your pet when he/she had his/her first fit?**  …… years …... months

**How many fits does your pet usually have in a 24hr period?** …………………………

**What is the most your pet has fitted in a 24hr period?**  …………………………

**If your pet fits more than once in a 24hr period, is this:**

once every month once every 3 months other frequency(specify) ..…………………………………………………………………………………………………………………………

**Has your pet ever had a status epilepticus (seizure lasting longer than 5 minutes) in his/her life?**

yes no
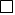
 not sure**
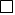
**

**How many fits does your pet have per month?** ………………………….

**If your dog is intact female, does the fit frequency increase during heat?**

yes no
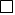
 not sure**
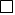
**

**Possible triggering events:**

**Do the fits occur:** in the morning in the afternoon at night any time

**Do the fits occur:** at rest during sleep during exercise/excitement

during stress soon after a meal when a meal is due

following exposure to flashing lights

following exposure to loud sounds

following any other stimulus (specify)……………………………………………………………..

**Before the fit:**

**Can you tell that your pet is going to have a fit before the event?** yes no not sure

**If yes, how long before the fit?** minutes hours days

**If yes, what signals the fit?**

fearfulness aggressiveness disorientation/agitation

wobbliness clinginess anti-social/hiding

sleepiness/lethargy staring into space fly-catching/star-gazing

sniffing

**Other:**

**The fit itself:**

**Do all the fits look the same?**  yes no not sure

Please describe the fit(s), elaborating on the above where appropriate. If your pet displays more than one type of fit, please describe the most common type first and then the other type/s:

**During the fit:**

**What is the very first thing that happens?** ..........................................................

………………………………………………………………………………………………………………………………

**Is the head involved first?** yes no not sure
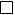


**Does your pet do chewing movements?** yes
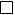
 no
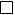
 not sure
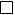


**Are hindlimbs or forelimbs involved first?** yes
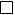
 no
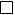
 not sure
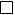


**Does the fit start on left or right side of your pet?** yes
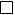
 no
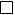
 not sure
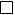


**Is your pet’s body stiff, floppy or normal?** stiff floppy
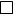
 normal

**Does your pet fall to the floor?**  yes no not sure

**If yes, is it always to the same side? yes, to the** ………… no

**Does your pet make chewing movements?**  yes no not sure

**Does your pet make paddling movements?**  yes no not sure

**Does your pet shake?**  yes no not sure

**Can your pet hear you?**  yes no not sure

**Can your pet see you?**  yes no not sure

**Does your pet:**  urinate defecate drool/froth

Please describe the fit(s), elaborating on the above where appropriate. If your pet displays more than one type of fit, please describe the most common type first and then the other type/s:

**How long do the fits last?**  …………………………

**Have you timed them with a watch?**  yes no

**How long does it take for your pet to be standing and walking around?** ……………………...

**How long does it take until your pet is back to normal?** …………………………

**After the fit:**

**Does your pet appear:**

fearful aggressive disorientated/agitated

wobbly/clumsy clingy anti-social/hiding

sleepy/lethargic staring into space fly-catching/star-gazing

sniffing blind normal

Other:

**Between fits:**

**Does your pet appear completely normal between events?** yes no not sure

**If no, how do they appear abnormal compared to before the fits started?**

**altered mentation? e.g. depressed, hyperactive** yes
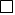
 no not sure

**unable to perform previously learned tasks?** yes no not sure

**abnormal social interaction? e.g. clingy, hiding** yes no not sure

**aggressive? e.g. towards dogs, towards people** yes no not sure

**disobedient?**  yes no not sure

**abnormal behaviour? e.g. staring into corners,** yes no not sure

**pressing head against walls**

**change in appetite** yes no not sure

**eating of excrement/abnormal items?** yes no not sure

**change in thirst?**  yes no not sure

**excessive licking or scratching themselves?**  yes no not sure

**destruction of property?**  yes no not sure

**difficulty settling? e.g. pacing, howling, barking**  yes no not sure

**change in sexual behaviour? e.g. towards humans,** yes no not sure **masturbation**

**change in sleeping pattern?** yes no not sure

**change in exercise pattern?**  yes no not sure

If you have any further information you would like to add, please do so here.

**If available, please provide us with:**

- **Video footage of the fit**
- **A copy of the seizure and medication diaries.**
- **Results of previous investigations (blood tests, urine test, MRI, CT, CSF analysis, others).**

**Thank you very much for your time.**
